# ALDOA inhibits cell cycle arrest induced by DNA damage via the ATM-PLK1 pathway in pancreatic cancer cells

**DOI:** 10.1186/s12935-021-02210-5

**Published:** 2021-09-26

**Authors:** Haidi Chen, Zeng Ye, Xiaowu Xu, Yi Qin, Changfeng Song, Guixiong Fan, Haifeng Hu, Yuheng Hu, Xianjun Yu, Wensheng Liu, Shunrong Ji, Wenyan Xu

**Affiliations:** 1 Department of Pancreatic Surgery, Fudan University Shanghai Cancer Center, No. 270 Dong’An Road, Shanghai, China; 2 Department of Oncology, Shanghai Medical College, Fudan University, Shanghai, China; 3 Shanghai Pancreatic Cancer Institute, No. 270 Dong’An Road, Shanghai, 200032 China; 4 Pancreatic Cancer Institute, Fudan University, Shanghai, China

**Keywords:** Cell cycle arrest, ALDOA, ATM, PLK1, Pancreatic cancer

## Abstract

**Background:**

ALDOA is a glycolytic enzyme found mainly in developing embryos, adult muscle and various malignant tumours, including pancreatic tumours. Our previous study revealed that ALDOA, an oncogene, can promote the proliferation and metastasis of pancreatic tumours. Furthermore, ALDOA could predict poor prognosis in patients with pancreatic tumours.

**Methods:**

IHC analysis of PDAC tissues was conducted. Western blotting, PCR, cellular IF experiments and cell cycle assessment were conducted utilizing cell lines. GSEA and KEGG pathway analysis were used to identify potential downstream pathways.

**Results:**

To explore the effects of ALDOA on the occurrence and development of pancreatic tumours, we analysed the RNA sequencing results and found that ALDOA could inhibit the DDR. Under normal circumstances, when DNA is damaged, initiation of the DDR causes cell cycle arrest, DNA repair or cell apoptosis. Further experiments showed that ALDOA could inhibit DNA repair and reverse cell cycle arrest induced by DNA damage so that DNA damage persisted to promote the occurrence and progression of cancer.

**Conclusions:**

Regarding the molecular mechanism, we found that ALDOA inhibited the DDR and improved activation of the cell cycle checkpoint PLK1 by suppressing ATM, which promotes tumour cell progression. Consequently, ALDOA has a profound effect on pancreatic cancer development.

## Introduction

Because of the presence of most of typical symptoms only at the middle and late stages, local recrudesce and distant metastasis and a poor response to almost all chemotherapy drugs, pancreatic cancer has a poor prognosis [1]. Although radical surgery is still the optimal curative option for patients with pancreatic tumours, the great majority of patients lose the opportunity for surgical excision due to late diagnosis [2]. Therefore, efforts to reveal the molecular and biological mechanisms of pancreatic cancer are urgently needed to discover more effective regimens for the comprehensive treatment of pancreatic cancer.

Fructose-bisphosphate aldolase A (ALDOA), a type of glycolytic enzyme, can catalyse reversible conversion, which converts fructose-1,6-bisphosphate to dihydroxyacetone phosphate and glyceraldehyde-3-phosphate [3]. ALDOA is extensively expressed in the vast majority of organs and tissues, primarily in the developing embryo and adult muscle, and supports the maintenance of a variety of biological processes and cellular functions related to muscle.[4] In addition, ALDOA overexpression has been discovered in various cancers, such as human colorectal cancer, lung squamous cell cancer, hepatocellular cancer, renal cell cancer and pancreatic cancer [4–6]. Our previous study indicated that ALDOA promoted the proliferation and metastasis of pancreatic cancer, partially through the regulation of E-cadherin expression, and predicted a dismal prognosis in patients with pancreatic cancer [7]. A recent study reported that increased transcript levels of ALDOA were correlated with the expression levels of cell cycle-related genes and that ALDOA is likely to affect cell cycle progression independent of glycolysis [3].

The DNA damage response (DDR), characterized by DNA repair and cell cycle arrest, is a highly conserved mechanism that occurs in response to genotoxic stress in cells that helps cells resist DNA damage induced by external and internal factors. Ataxia telangiectasia mutated (ATM), the most upstream DDR kinase, is able to identify DNA damage, transmit DNA damage signals to downstream target proteins, activate stress systems, and ultimately induce cell cycle arrest [8–10]. Gaul conducted in vitro experiments and confirmed that bendamustine could induce G2 phase arrest by activating the ATM-Chk2-Cdc25 signalling pathway in myeloma cells [11]. Therefore, ATM is crucial to maintaining the stability of the cell genome and preventing tumour occurrence.

Polo-like kinase 1 (PLK1), an essential cell cycle regulator and a member of the serine/threonine-protein kinase family, is overexpressed in various human cancers. A novel study provided in vivo evidence that PLK1 induces chromosomal instability and overrides cell cycle checkpoints to drive tumorigenesis [12]. In another study, it was reported that RO3280 (a PLK1 inhibitor) restrained the proliferation of cancer cells by inducing cell cycle arrest at the G2/M point [13]. These findings indicate the association between ATM and PLK1. However, the effect of PLK1 on the DDR has not been fully investigated. Moreover, few studies have explored the relationship between ALDOA and PLK1. Hence, it is important to investigate the effects of PLK1, ATM and ALDOA on the DDR and the potential value of these proteins as targets in cancer therapy. Both PLK1 and ATM regulate the cell cycle, and it is important to determine whether ALDOA, which connects ATM to PLK1, regulates the cell cycle arrest caused by DNA damage and whether this regulatory mechanism is significant in the development of pancreatic cancer.

In our research, we probed the potential role of ALDOA in the cell cycle arrest induced by DNA damage in pancreatic tumour cells and discovered that ALDOA participated in regulating the DDR and attenuated the cell cycle arrest induced by DNA damage. Regarding the pathway, ALDOA increased PLK1 activation by suppressing ATM expression to alter the induction of cell cycle arrest by DNA damage. Further studies revealed that ALDOA expression positively correlates with PLK1 expression and negatively correlates with ATM expression in pancreatic cancer patients.

## Methods

### Cell culture

The human pancreatic cancer cell lines SW1990 and PANC-1 were acquired from the American Type Culture Collection (ATCC, USA) and cultured at 5 % CO_2_ and 37 °C in Dulbecco’s modified Eagle’s medium (DMEM) containing 10 % FBS.

### RNA isolation and quantitative real-time PCR

Total RNA was extracted by using TRIzol reagent (Invitrogen, Carlsbad, CA, USA). Quantitative real-time PCR was performed as previously described [7]. The expression levels of β-actin and the target genes were confirmed with an ABI 7900HT Real-Time PCR system (Applied Biosystems, USA). The ΔΔCt method was used to quantify the expression of target genes, and each reaction was conducted three times.

The following primers were used:

Human ALDOA: 5’-GTTATCAAATCCAAGGGCGGTGTT-3’ (forward).

5’-AGTCAGCTCCGTCCTTCTTGTAC-3’ (reverse).

Human ATM: 5’-CCGAGTGCAGTGACAGTGAT-3’ (forward).

5’-TTGACGGCAGCAGATAAGCA-3’ (reverse).

Human β-actin: 5’-CTACGTCGCCCTGGACTTCGAGC-3’ (forward).

Human ATR: 5’-GGAGATTTCCTGAGCATTCGAGC-3’ (forward).

5’-GGCTTCTTTACTCCAGACCAATC-3’ (reverse).

Human NBN: 5’-TCTGTCAGGACGGCAGGAAAGA-3’ (forward).

5’-CACCTCCAAAGACAACTGCGGA-3’ (reverse).

Human RBBP8: 5’-TGGCAGACAGTTTCTCCCAAGC-3’ (forward).

5’-GGCTCCACAAACGCTTTCTGCT-3’ (reverse).

Human RAD17: 5’-GGTCCAAGCTATTGGTGGCAAAG-3’ (forward).

5’-AATGAGAGGGCAACCGAGGTGA-3’ (reverse).

Human BLM: 5’-GGTGATAAGACTGACTCAGAAGC-3’ (forward).

5’-AACGTGCCAAGAGCTTCCTCTC-3’ (reverse).

Human LIG4: 5’-CAGCAGAGATCGTACCCAGTGA-3’ (forward).

5’-TGCGAGCTTACCAGATGCCTTC-3’ (reverse).

Human POLK: 5’-CAATGCCCAGTGGCAAACCTCA-3’ (forward).

5’-ACGCAGTTCCTTCTACACCAGC-3’ (reverse).

Human BRCA2: 5’-GGCTTCAAAAAGCACTCCAGATG-3’ (forward).

5’-GGATTCTGTATCTCTTGACGTTCC-3’ (reverse).

### Protein extraction and western blot analysis

Western blotting was performed as previously described [7]. ALDOA (A1142), ATM (A5908), PLK1 (A2548), phospho-PLK1-T210 (AP0519) and β-actin (AC026) antibodies were purchased from ABclonal.

### Small compounds

Cisplatin (HY-17,394), temozolomide (HY-17,364) and KU-55,933 (HY-12,016) were purchased from MedChemExpress.

### Colony formation assay

Two hundred cells were seeded into a new 6-well plate and cultured for 2 weeks to allow colony formation. The cells were then fixed using methanol, stained using 0.1 % crystal violet solution and finally counted.

### Lentivirus production

The pLKO.1-TRC cloning vector (Addgene Plasmid 10878) was used to generate short hairpin RNA (shRNA) constructs to silence ALDOA expression. The sequences (21 bp) against ALDOA were 5’- CCATGCTTGCACTCAGAAGTT-3’ and 5’-TGGCGTTGTGTGCTGAAGATT-3’. pLKO.1-sh-scramble (Addgene plasmid 1864) was used as a control plasmid. To obtain ALDOA overexpression constructs, FLAG-tagged ALDOA was cloned into pCDH-CMV-MCS-EF1-Puro vector, and empty vector (EV) was used as the control. Lentiviral particles were produced by cotransfection of pLKO.1-shALDOA- or ALDOA-expressing constructs with psPAX2 and pMD2.G into HEK-293T cells. Lentiviral particles were harvested, filtered, and applied to infect SW1990 and PANC-1 cells, which were then subjected to puromycin selection. ATM-specific small interfering RNA (siRNA), PLK1‐specific siRNA, negative control siRNA, PCMV-PLK1-FLAG plasmid and PCMV-C-FLAG plasmid were transfected into pancreatic cancer cells by applying Lipofectamine™ 3000 (Invitrogen). The following siRNA sequences were used:

ATM-specific siRNA: 5’-GGCACUUUGUGAUGCUUAUdTdT-3’ (forward).

5’-AUAAGCAUCACAAAGUGCCdTdT-3’ (reverse).

PLK1-specific siRNA: 5’-CUAAGUCUCUGCUGCUCAAdTdT-3’ (forward).

5’-UUGAGCAGCAGAGACUUAGdTdT-3’ (reverse).

### Cell cycle assessment

Cells were collected, fixed with 70% alcohol at 4 °C for half an hour, and then washed twice with cold phosphate-buffered saline (PBS). Then, a mixture of RNaseA (Beyotime), propidium iodide (PI, Beyotime) and staining buffer (Beyotime) was added to the cells, which were incubated at 37 °C for half an hour in the dark following the manufacturer’s instructions. A FACSCalibur flow cytometer (BD, USA) was used to determine the cell cycle distribution. The results were obtained using MultiCycle software (Beckman Coulter, USA).

### Cellular immunofluorescence (IF)

The cells mounted on slides were fixed with 4% paraformaldehyde at 4 °C, permeabilized with 0.2% Triton X-100 and subsequently blocked with 5% bovine serum albumin buffer at 37 °C for one hour. The cells were then were incubated with phospho-H2AX-S139 (γ-H2AX, ab81299, Abcam) antibodies at 4 °C overnight and FITC (AS011, ABclonal) antibodies at 37 °C for 1 h. DAPI (Sigma) was used to stain nuclear DNA. A Leica TCS SP5 confocal microscope was used to obtain images.

### Immunohistochemistry (IHC) staining

In this study, clinical tissue samples were obtained from patients who had been diagnosed with pancreatic cancer at Fudan University Shanghai Cancer Center; sampling was conducted with patient consent and approval from the Institutional Research Ethics Committee. Rigorous diagnoses were established by 2 skilled pathologists. IHC staining of ALDOA, ATM and PLK1 was conducted in accordance with standard procedures, as described previously [7]. Anti-ALDOA antibody (A11445; ABclonal), anti-ATM antibody (A19650; ABclonal) and anti-PLK1 antibody (A2548; ABclonal) were each used at a dilution factor of 1:100. Under microscope, three different fields of view for every slide were selected randomly for scoring. The staining intensity and proportion of positively stained cells were quantitatively scored. The levels of protein expression were calculated by multiplying the proportion score (0, 0–5%; 1, 6–25%; 2, 26–50%; 3, 51–75%; and 4, 76–100 %) and the intensity score (0, no colouration; 1, pale yellow; 2, clay bank; and 3, brown).

### Statistical analysis

Data are presented as the mean ± SD. All statistical analyses were performed with GraphPad Prism (GraphPad Software, USA), and one-way ANOVA and Student’s t-test were performed to compare the data between groups. The correlations among ALDOA, ATM and PLK1 expression were analysed using Spearman correlation analysis. Differences were considered significant at *P < 0.05; **P < 0.01, ***P < 0.001.

## Results

### ALDOA participated in the regulation of the cell cycle and the DDR

**Fig. 1:**
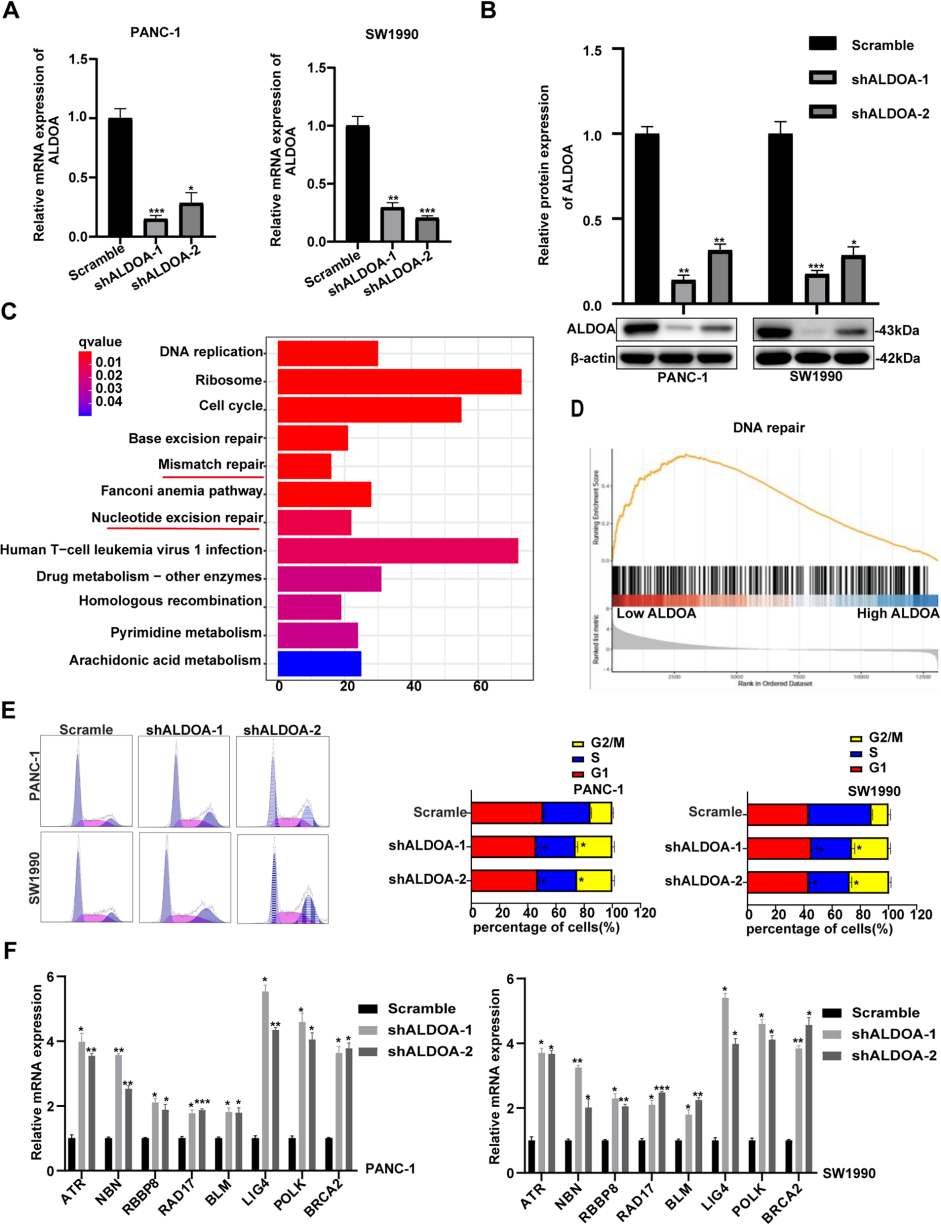
ALDOA participated in the regulation of the DDR. **A** qRT-PCR analysis of the knockdown efficiency of shALDOA. **B** Western blotting was applied to analyse the knockdown efficiency of shALDOA. **C** KEGG analysis of the differentially expressed genes from the RNA sequencing conducted on PANC-1 and SW1990 cells showed the core enriched signalling pathways in ALDOA-silenced cells versus NC cells. **D** GSEA was applied to analyse the signalling pathway enrichment in siALDOA versus siNC PANC-1 and SW1990 cells. **E** Cell cycle analysis via flow cytometry revealed obvious arrest in the G2/M phase. **F** qRT-PCR analysis of the expression of genes related to DNA repair

By shRNA-mediated silencing with two shRNA constructs, we reduced ALDOA expression in the PANC-1 and SW1990 cell lines. The knockdown efficiency was confirmed by western blotting and quantitative RT-PCR (Fig. 1A and B). RNA sequencing was performed in PANC-1 and SW1990 cell lines with scramble and knockdown ALDOA. Subsequently, we selected genes expressed at less than 0.5-fold or greater than 1.5-fold in the low ALDOA expression group relative to the scramble group. To identify correlated pathways and genes, gene set enrichment analysis (GSEA) and Kyoto Encyclopedia of Genes and Genomes (KEGG) pathway analysis were conducted, and the results revealed that ALDOA might be related to DNA repair (including mismatch repair and nucleotide excision repair) (Fig. 1C and D). Our previous results showed that ALDOA promoted proliferation and metastasis in pancreatic cancer cells [3, 7]. Here, we found that the expression of ALDOA and some genes related to DNA repair followed opposite trends, indicating that ALDOA might inhibit DNA repair. DNA damage often leads to cell cycle arrest or apoptosis. If ALDOA inhibits DNA repair, then ALDOA can be expected to induce cell cycle arrest, which seems to contradict our previous research. To further explore the relationship between ALDOA and DNA damage as well as the cell cycle, we first tested the influence of ALDOA knockdown on the cell cycle through flow cytometry and found that ALDOA knockdown caused cell cycle arrest (Fig. 1E). Subsequently, we verified the expression of genes related to DNA repair in the sequencing results following ALDOA knockdown through PCR and found that ALDOA knockdown promoted the expression of these genes, which suggested that ALDOA might inhibit the regulation of DDR but attenuate the cell cycle arrest induced by DNA damage (Fig. 1F).

### ALDOA inhibited the cell cycle arrest induced by DNA damage

**Fig. 2:**
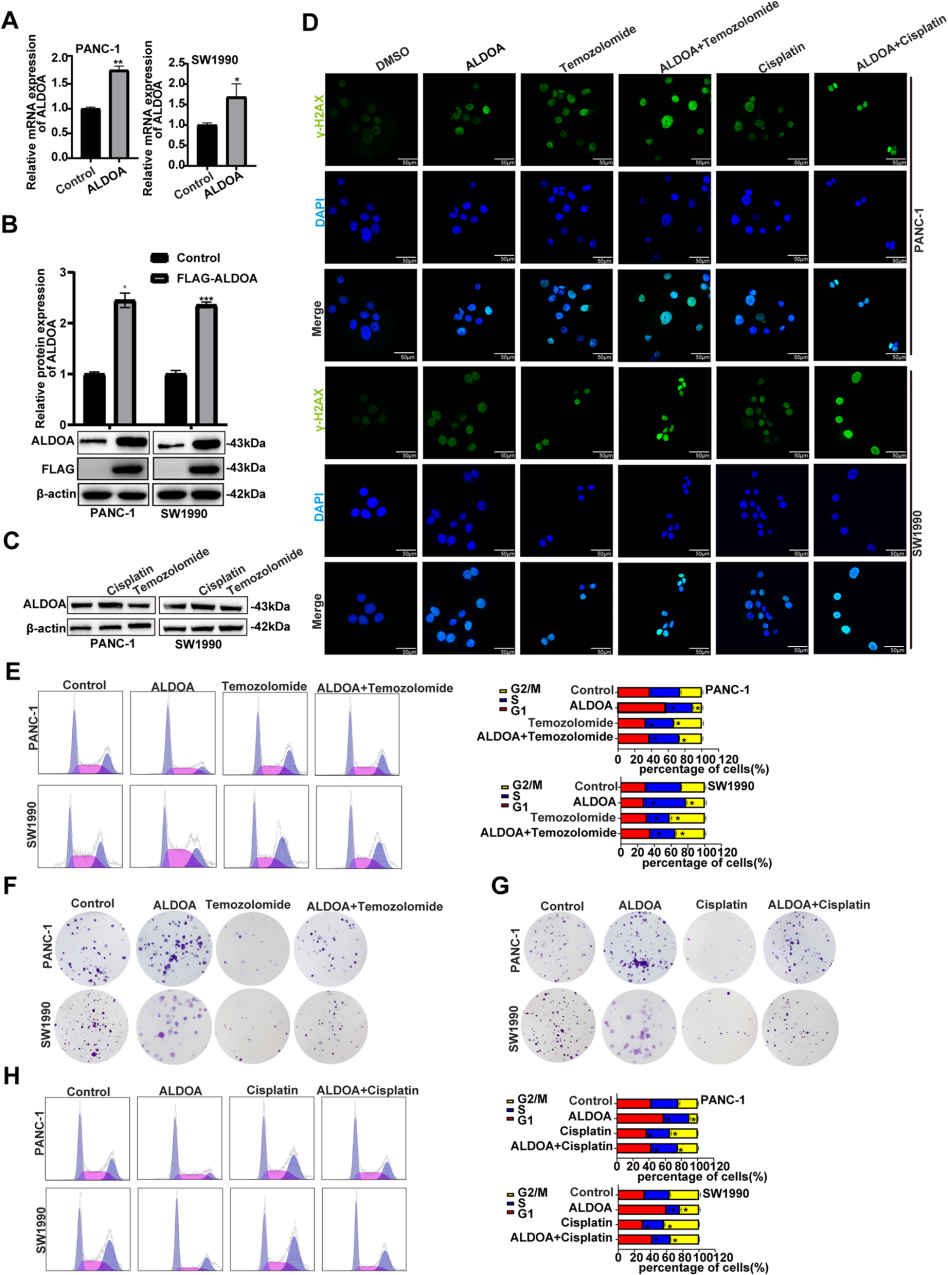
ALDOA inhibited cell cycle arrest induced by DNA damage. **A** and **B** Western blot and qRT-PCR assays confirmed the efficiency in ALDOA-overexpressing PANC-1 and SW1990 cells. **C** Cell lines were treated with cisplatin (15 µM)/temozolomide (150 µM) to assess the protein level of ALDOA. **D** Confocal microscopy showed DNA damage (green) and nuclear DNA (blue) in ALDOA-overexpressing or normal PANC-1 and SW1990 cells treated with cisplatin (15 µM)/temozolomide (150 µM). **E** and **H** Cell cycle analysis by flow cytometry indicated a marked change in the proportion of cells in G2/M phase in ALDOA-overexpressing or normal PANC-1 and SW1990 cells treated with cisplatin (15 µM)/temozolomide (150 µM). **F** and **G** The ALDOA group and the control group respectively were treated with cisplatin (15 µM)/temozolomide (150 µM) to assess colony formation capacity

To verify that ALDOA could reverse the cell cycle arrest caused by DNA damage, we increased ALDOA expression via a pCDH-CMV-MCS-EF1-Puro vector with an ALDOA construct, and we validated the overexpression efficiency by western blotting and quantitative RT-PCR (Fig. 2A and B). We used cisplatin and temozolomide to imitate the DNA damage environment, and we stained for γ-H2AX, a sensitive marker of DNA damage, to accurately assess DNA damage. At the same time, we verified that DDR did not affect ALDOA expression by western blotting, which ruled out the effect of DDR on ALDOA status (Fig. 2C). Images of the immunofluorescence assay (IFA) obtained under a copolymerization microscope clearly showed an increase in γ-H2AX level after treatment with ALDOA, cisplatin and temozolomide (Fig. 2D). Interestingly, we found that, treated with cisplatin and temozolomide, the ALDOA group possessed a lower percentage of cells in G2/M phase and stronger colony formation capacity than the control group (Fig. 2E, F, G and H). These results indicated that ALDOA might inhibit the cell cycle (G2 phase) arrest induced by DNA damage.

### ALDOA decreased the percentage of cells in G2/M phase by suppressing ATM expression

**Fig. 3:**
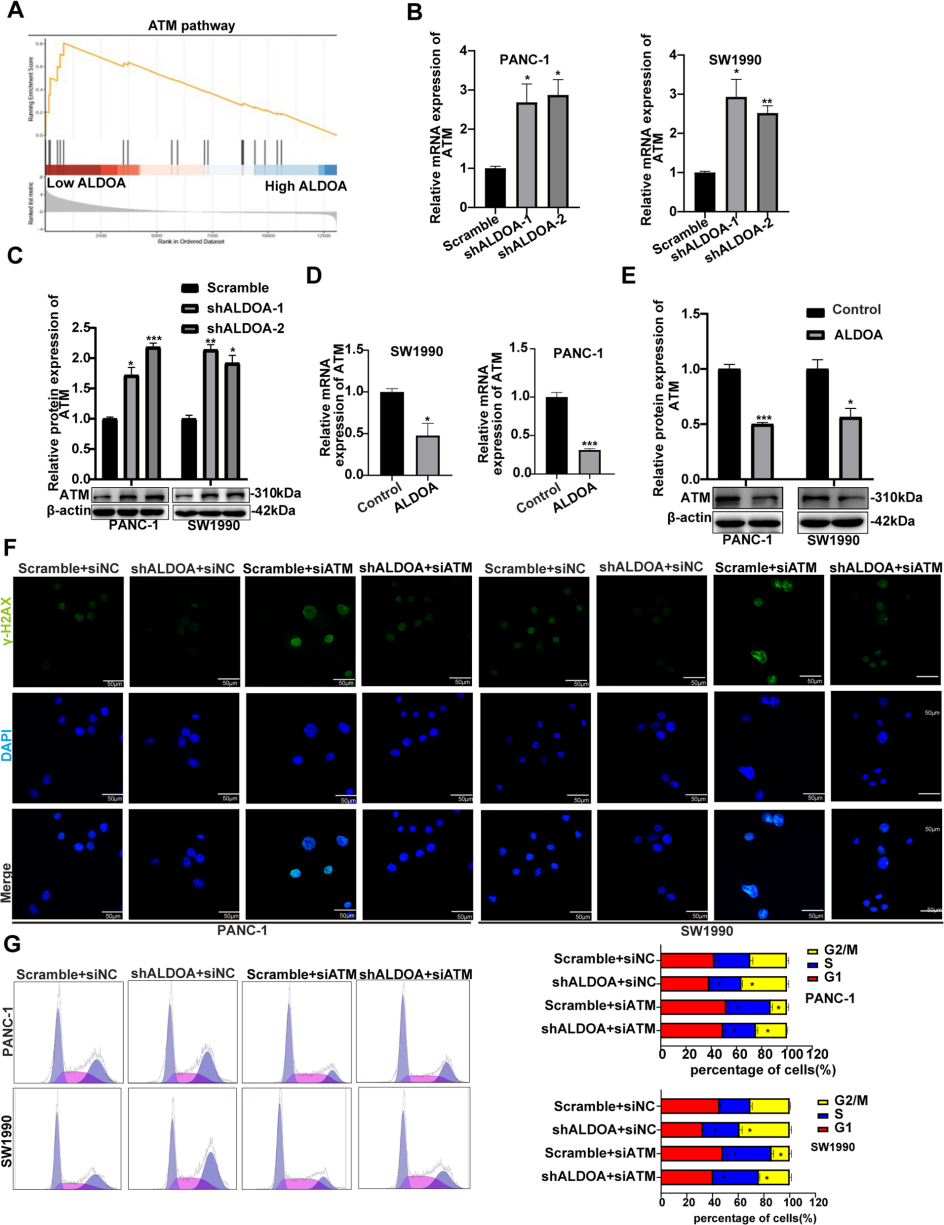
ALDOA decreased the percentage of cells in G2/M phase by suppressing ATM expression. **A** GSEA was conducted to analyse the signalling pathway enrichment in siALDOA PANC-1 and SW1990 cells compared with the NC group. **B**, **C**, **D** and **E** The mRNA and protein levels of ATM expression in ALDOA-silenced and ALDOA-overexpressing cell lines (PANC-1 and SW1990). **F** Confocal microscopy showed DNA damage (green) and nuclear DNA (blue) in ALDOA-silenced or normal cells (PANC-1 and SW1990) treated with siATM or siNC. **G** Cell cycle analysis by flow cytometry demonstrated an obvious change in the proportion of cells in G2/M phase in ALDOA-silenced or normal cells (PANC-1 and SW1990) treated with siATM or siNC

We analysed the GSEA results again to further explore the molecular mechanism by which ALDOA reverses the cell cycle arrest caused by DNA damage and found that ALDOA and ATM are closely related (Fig. 3A). ATM is a key molecule in the DDR and plays a significant role in damage repair and cell cycle regulation. To assess whether ALDOA alters the percentage of cells in G2/M phase via ATM, we assessed the protein and mRNA levels of ATM in ALDOA-silenced and ALDOA-overexpressing PANC-1 and SW1990 cell lines. Our data indicated that ATM protein and mRNA levels increased significantly when ALDOA expression was silenced (Fig. 3B, C), while ALDOA overexpression yield the opposite results (Fig. 3D, E). To verify the role of ATM in the ALDOA-induced cell cycle progression, we transfected ATM siRNA into pancreatic cancer cells stably expressing ALDOA shRNA vector or scramble vector and found that transfection reduced the percentage of cells in G2/M phase in the ALDOA shRNA vector cells compared with the cells expressing scramble vector. We observed that ATM silencing dramatically reduced the increase in the percentage of cells in G2/M phase caused by silenced ALDOA expression (Fig. 3G). These results suggest that ALDOA might reduce the percentage of pancreatic cancer cells in the G2/M phase by suppressing ATM expression. To estimate the effect of ATM on DNA damage, IFA was conducted, and the images indicated that the scramble- and shALDOA-transfected cells that were cotransfected with ATM siRNA displayed higher γ-H2AX levels than those cotransfected with siNC(Fig. 3F). These results indicate that ALDOA can affect DNA repair and the cell cycle by inhibiting ATM.

### ALDOA promoted PLK1 activation by suppressing ATM expression

**Fig. 4:**
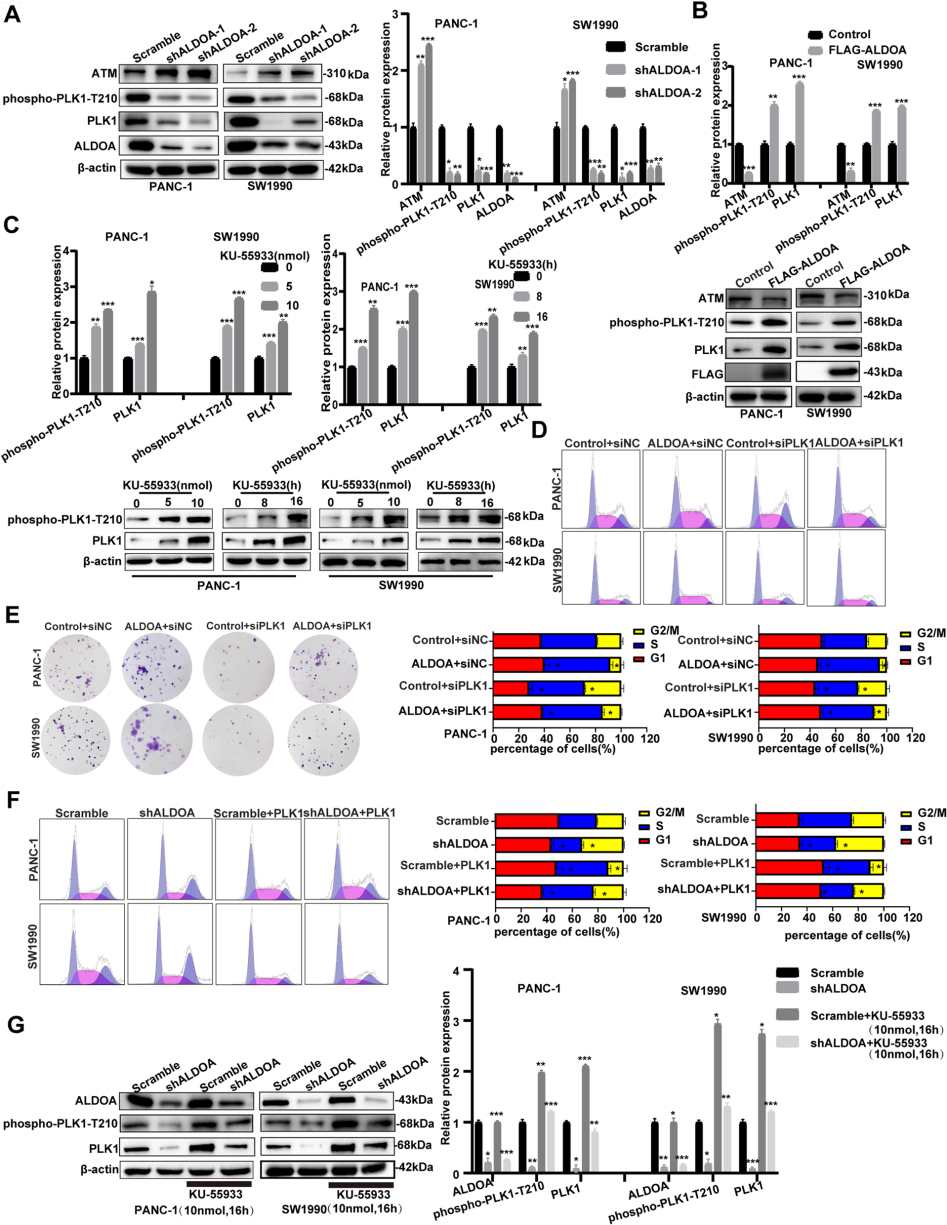
ALDOA promoted PLK1 activation by suppressing ATM expression. **A** and **B** The expression of ATM, phospho-PLK1-T210, PLK1 and ALDOA was determined by western blot analysis. **C** Dose-dependent effect of KU-55,933 treatment for 16 h on the phospho-PLK1-T210 and PLK1 protein. The expression of phospho-PLK1-T210 and PLK1 in PANC-1 or SW1990 cells treated with 10 ng/ml KU-55,933 over a time gradient. **D** and **F** PLK1 exerted significant regulation of the G2/M phase in cell cycle analysis by flow cytometry. E. The ALDOA group and the control group respectively were transfected by siPLK1 and siNC to assess colony formation capacity. **G** The expression of ALDOA, phospho-PLK1-T210 and PLK1 was determined by western blot analysis

**Fig. 5:**
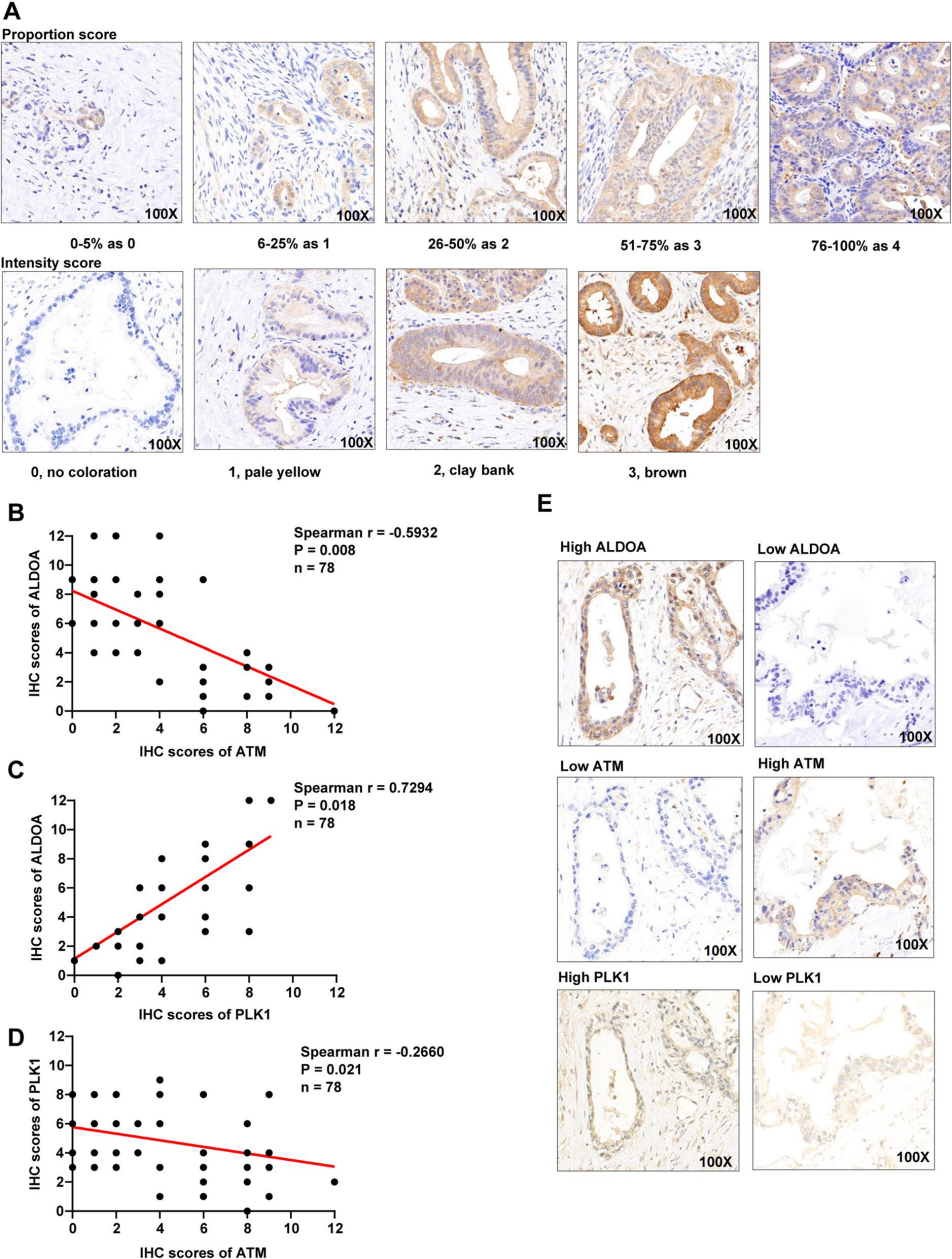
ALDOA expression positively correlates with PLK1 expression and negatively correlates with ATM expression in clinical samples. **A** Micrographs showing the proportion score and intensity score of ALDOA, ATM and PLK1. **B** ATM expression was negatively correlated with ALDOA expression in clinical samples, as shown in IHC. **C** PLK1 expression was positively correlated with ALDOA expression in clinical samples, as shown in IHC. **D** ATM expression was negatively correlated with PLK1 expression in clinical samples, as shown in IHC. **E** Clinical samples with higher levels of ALDOA revealed lower ATM expression and higher PLK1 expression

ATM has been reported to influence the cell cycle through regulation of PLK1, and PLK1 is the G2/M checkpoint that alleviates cell cycle arrest in the G2/M phase in various tumour cells. Mutational analysis confirmed that threonine 210 (T210), located in the activation loop of the kinase domain, is the chief activation site of PLK1. Therefore, we suspected that phospho-PLK1-T210 might participate in the cell cycle arrest caused by ALDOA and ATM. To probe the mechanism, we investigated the protein levels of ATM, PLK1 and phospho-PLK1-T210, vital regulators of the DDR, to determine the intrinsic link between these proteins and ALDOA. Silencing ALDOA decreased PLK1 and phospho-PLK1-T210 protein levels but increased ATM protein levels (Fig. 4A). We observed the reverse trends when ALDOA was overexpressed (Fig. 4B). PLK1 activation was restrained by ATM/ATR-dependent checkpoint pathways in response to DNA damage. Moreover, Tsvetkov et al. indicated that phosphorylation of PLK1 at T210 (phospho-PLK1-T210) was inhibited by DNA damage. Next, we administered phospho-PLK1-T210 to determine whether ATM inhibits PLK1 activation in human pancreatic cancer cells. KU-55,933 (an ATM kinase inhibitor) has been reported to be an effective, specific ATM inhibitor. As displayed in Fig. 4C, KU-55,933 promoted increases in PLK1 and phospho-PLK1-T210 levels in a time- and dose-dependent manner (Fig. 4C). Therefore, ATM might restrain PLK1 activation to form phospho-PLK1-T210. To identify the function of PLK1 in the ALDOA-induced cell cycle, PLK1 siRNA was transfected into SW1990 and PANC-1 cells stably overexpressing ALDOA, and we discovered that PLK1 silencing notably offset the inhibition of cell cycle arrest in the G2/M phase and the increase in colony formation capacity caused by ALDOA overexpression (Fig. 4D and E). Furthermore, we transfected the PCMV-PLK1-FLAG plasmid into pancreatic cancer cells with stably silenced ALDOA. The results revealed that increasing PLK1 markedly neutralized the decrease in the proportion of cells arrested in the G2/M phase caused by ALDOA knockdown (Fig. 4F). Based on the above results, we speculated that ALDOA might reduce the cell cycle arrest in G2/M phase by positive regulation of PLK1 expression. Therefore, we next used KU-55,933 to inhibit ATM in ALDOA knockdown cells and scramble cells and investigated the changes in the expression levels of related proteins caused by ATM. The results demonstrated that KU-55,933 could reverse the changes in PLK1 and phospho-PLK1-T210 protein expression caused by ALDOA knockdown but did not change ALDOA expression (Fig. 4G). These results indicate that ALDOA might activate PLK1 by decreasing the ATM protein level (Fig. 5).

### ALDOA expression positively correlates with PLK1 expression and negatively correlates with ATM expression in pancreatic cancer patients

To verify the state of expression of ALDOA, ATM and PLK1 in patient tissues, we randomly selected 78 patients diagnosed with pancreatic cancer in our centre. Next, we conducted IHC staining of pancreatic ductal adenocarcinoma (PDAC) tissues using antibodies against ALDOA, ATM and PLK1. Furthermore, we calculated the IHC score by multiplying the intensity score and the proportion score. The scoring parameters are shown in Fig. 5A. We then analysed the pairwise correlations among ALDOA, ATM and PLK1 and found significant negative correlations between ALDOA and ATM and between ATM and PLK1 but a positive correlation between ALDOA and PLK1 in pancreatic cancer patients (Fig. 5B, C, D). Figure 5E shows two typical examples of the same trend in expression levels.

**Fig. 6:**
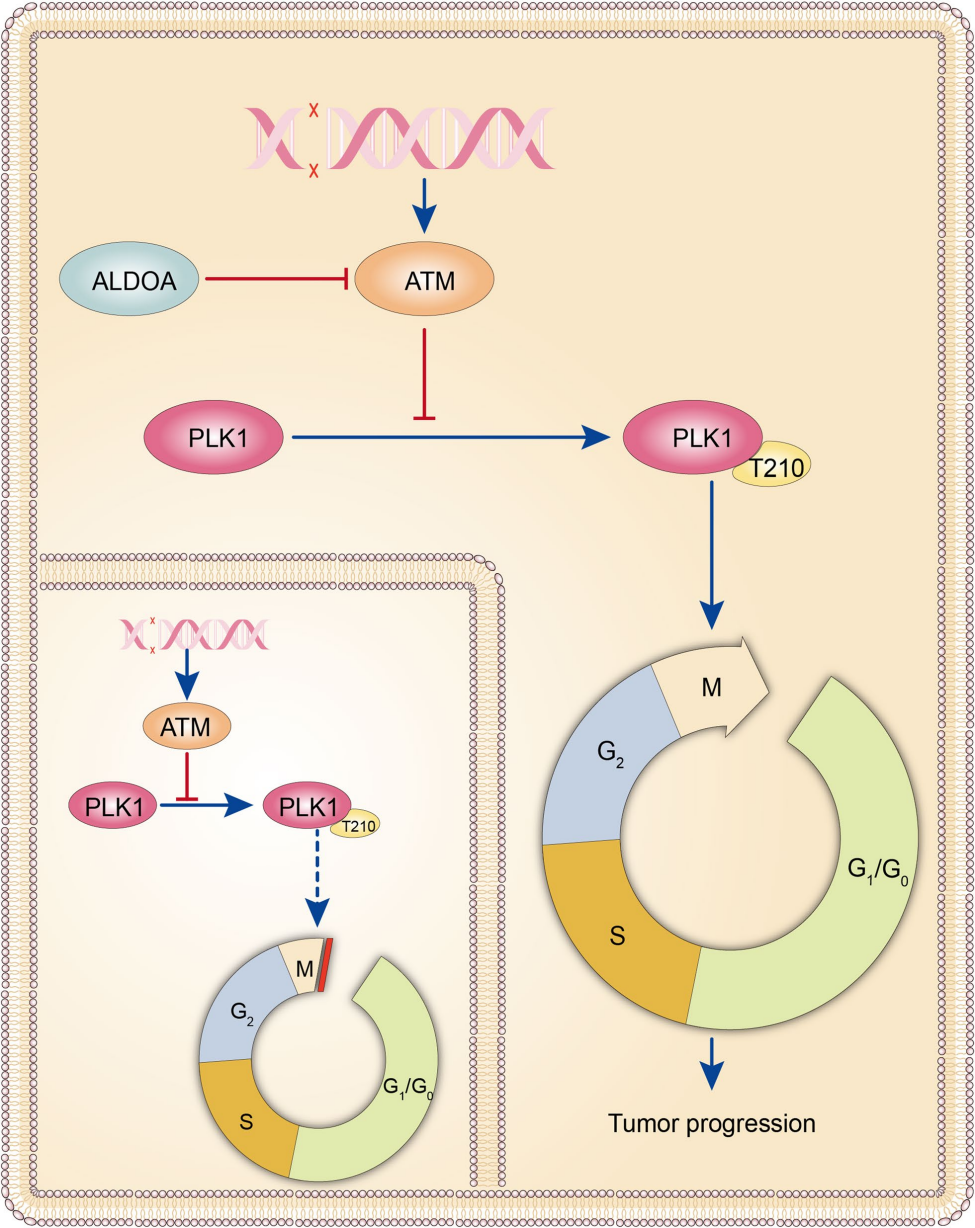
Schematic illustration depicting the function of ALDOA: ALDOA reverses the cell cycle arrest induced by DNA damage by inhibiting ATM and promoting PLK1 activation. The dashed arrow highlights the alleviation of cell cycle arrest in G2/M phase by phospho-PLK1-T210 in tumour cells without ALDOA

## Discussion

The DDR, which involves cell cycle arrest, DNA repair, and apoptosis, is the response of cells to genotoxic stress. When the DDR pathway is intact, the genetic material of eukaryotic cells can be replicated steadily and passed exactly to the next generation. ATM can identify DNA damage, transmit relevant signals to downstream target proteins, activate the stress system, induce cell cycle arrest, and facilitate DNA repair or initiate cell apoptosis [10, 14]. Therefore, ATM is essential to maintaining the stability of the cell genome and preventing the occurrence of tumours. Gaul conducted in vitro experiments to identify the molecular mechanism of action of bendamustine, which can kill cancer cells, and found that bendamustine, as an ATM promoter, can induce G2 cell cycle arrest [11]. PLK1, a member of the serine/threonine-protein kinase family, is a key regulator of nonmitotic processes and phenomena, such as DNA replication, DDR and G2 DNA damage checkpoint recovery, chromosome dynamics, and microtubule dynamics, and it is activated by phosphorylation at the G2/M phase boundary [15]. A study by Lilia Gheghiani provides in vivo proof that abnormal expression of PLK1 triggers the overriding of cell cycle checkpoints to drive tumorigenesis and chromosomal instability and that high expression levels of PLK1 are always correlated with poorer patient prognosis and are related to high tumour grade, which strongly indicates that PLK1 plays key roles in tumour progression and initiation [12, 16, 17]. Furthermore, PLK1 activity is inhibited by ATM/ATR-dependent checkpoint pathways during the DDR, and phosphorylation of PLK1 at T210, the major activation site of PLK1, is a potential target of DNA damage checkpoints [18].

ALDOA, one of the glycolytic enzymes, has complex and important functions in cells. However, the relationship between ALDOA and DDR-induced cell cycle arrest is unclear. In this study, we found that ALDOA participated in the regulation of the DDR and restored the cell cycle arrest caused by DNA damage. The underlying mechanism is not clear. Perhaps there is a pathway activated in pancreatic cancer that can change the cell cycle process independent of glycolysis [19, 20]. However, further study is needed for verification. We also revealed that ALDOA reduced the percentage of cells in G2/M phase by suppressing the expression of ATM, which is one of the most upstream DDR kinases. Further study showed that ALDOA promoted PLK1 activation by suppressing ATM expression. Intriguingly, ATM has been demonstrated to inhibit PLK1 activity during the DDR [21]. These results indicate that ALDOA might inhibit the cell cycle arrest induced by DNA damage via the ATM-PLK1 pathway in pancreatic cancer cells. In summary, ALDOA inhibits DNA repair but releases the cell cycle arrest induced by DNA damage, which allows cells to continue to expand and DNA damage to remain and accumulate, induces genic mutations and promotes the occurrence and development of tumours. We also found an interesting phenomenon in which ALDOA aggravated DNA damage but reversed the cell cycle arrest induced by DNA damage, and we discovered that DNA damage might be relieved by ATM. Therefore, this phenomenon might be caused by ALDOA-mediated inhibition of ATM.

A limitation of this research is that we did not further illustrate how ALDOA controls cell cycle arrest induced by the DDR via the ATM-PLK1 pathway. Through its impact on aerobic glycolysis, ALDOA can cause imbalance in the cancer microenvironment, and accumulating evidence has shown that ALDOA promotes proliferation and metastasis via its nonenzymatic functions in tumour cells [22]. Through a variety of processes that affect cell proliferation, ALDOA has been confirmed to translocate to the nucleus [20, 23]. This discovery indicates that ALDOA has the ability to influence gene transcription. Therefore, we speculate that ALDOA might translocate to the nucleus to function as a transcription factor and thereby affect ATM expression.

In conclusion, this study reveals the role of ALDOA in cell cycle arrest induced by the DDR and provides a preliminarily exploration of the underlying regulatory pathways (Fig. 6).

## Data Availability

The data that support the findings of this study are available from the corresponding author upon reasonable request.
